# Identification of CiaR Regulated Genes That Promote Group B Streptococcal Virulence and Interaction with Brain Endothelial Cells

**DOI:** 10.1371/journal.pone.0153891

**Published:** 2016-04-21

**Authors:** Rong Mu, Andrew S. Cutting, Yvette Del Rosario, Nicholas Villarino, Lara Stewart, Thomas A. Weston, Kathryn A. Patras, Kelly S. Doran

**Affiliations:** 1 Department of Biology and Center for Microbial Sciences, San Diego State University, San Diego, California, 92182, United States of America; 2 Department of Pediatrics, University of California San Diego School of Medicine, La Jolla, California, 92093, United States of America; Hungarian Academy of Sciences, HUNGARY

## Abstract

Group B *Streptococcus* (GBS) is a major causative agent of neonatal meningitis due to its ability to efficiently cross the blood-brain barrier (BBB) and enter the central nervous system (CNS). It has been demonstrated that GBS can invade human brain microvascular endothelial cells (hBMEC), a primary component of the BBB; however, the mechanism of intracellular survival and trafficking is unclear. We previously identified a two component regulatory system, CiaR/H, which promotes GBS intracellular survival in hBMEC. Here we show that a GBS strain deficient in the response regulator, CiaR, localized more frequently with Rab5, Rab7 and LAMP1 positive vesicles. Further, lysosomes isolated from hBMEC contained fewer viable bacteria following initial infection with the Δ*ciaR* mutant compared to the WT strain. To characterize the contribution of CiaR-regulated genes, we constructed isogenic mutant strains lacking the two most down-regulated genes in the CiaR-deficient mutant, SAN_2180 and SAN_0039. These genes contributed to bacterial uptake and intracellular survival. Furthermore, competition experiments in mice showed that WT GBS had a significant survival advantage over the Δ*2180* and Δ*0039* mutants in the bloodstream and brain.

## Introduction

Bacterial pathogens that have the capability of penetrating the central nervous system (CNS) thereby eliciting life-threatening diseases are a major human health concern. A severe outcome of bacterial infiltration of the CNS is the development of meningitis. One such pathogen, Group B *Streptococcus* (GBS), is a Gram-positive bacterium that is the leading cause of neonatal meningitis. Although intrapartum chemoprophylaxis is available to pregnant mothers during delivery, GBS infections among both pre-term and term infants still occurs [1]. Infants who survive meningitis suffer long-term neurological complications including developmental delays, hydrocephalus, visual impairment, deafness, cerebral palsy, and seizures [2]. GBS-induced meningitis occurs upon blood-brain barrier (BBB) penetration after a prolonged period of bacteremia [3]. Persistent blood-borne bacteria evade a variety of host defenses and have the propensity to cross the BBB, although the exact mechanism(s) of GBS-BBB transit are still being discovered. The majority of the BBB is composed of a specialized single cell layer known as human brain microvascular endothelial cells (hBMEC), which regulates passage of molecules, nutrients, and infectious agents into the brain [4]. Still, a few bacterial pathogens like GBS are able to disrupt this barrier to gain access to the CNS, resulting in inflammation, BBB permeability, and disease progression.

Much research has been devoted toward understanding the key GBS virulence factors that allow for BBB transit and breakdown. It is believed that direct invasion and subsequent transcytosis of brain endothelial cells by GBS is the critical first step for the development of meningitis [5]. Our lab has published several studies implicating multiple bacterial factors that participate in this initial invasion processes into brain endothelium including GBS surface associated proteins such as pili, lipoteichoic acid (LTA), serine rich repeat (Srr) proteins and a fibronectin binding protein, SfbA [6–10]. Following bacterial uptake, electron microscopy (EM) studies have demonstrated the presence of GBS in membrane-bound vesicles within hBMEC [11,12] suggesting the involvement of endocytic pathways, however, little is known about how GBS persists and traffics through the BBB. We have recently demonstrated that autophagy is induced in BBB endothelium during GBS infection, but that this pathway was not effective in completely eliminating intracellular GBS [12]. Thus, an understanding of how GBS resists intracellular host defenses and transits through brain endothelial cells is warranted.

To this end, we have investigated GBS trafficking within brain endothelial cells and the bacterial factors responsible for GBS survival. Endocytic trafficking is initiated upon bacterial invasion of host cells and subsequently, Rab GTPases aide in delivering these invaders to the lysosome for degradation [13]. Numerous bacterial pathogens, such as *Legionella pneumophila*, *Mycobacterium tuberculosis*, *Pseudomonas aeruginosa*, and *Salmonella enterica* are known to inhibit or disrupt endocytic trafficking to establish an intracellular niche or simply promote survival or growth [14]. To accomplish this, bacteria likely modulate gene expression to adapt to different host cellular environments, often through two component regulatory systems (TCRS). TCRS function through phosphotransfer signals from a membrane-bound sensor histidine kinase, which senses environmental changes, to subsequent activation of a cytoplasmic response regulator, with downstream transcription modulation [15]. GBS genome sequence analysis suggests multiple putative TCRS, but most of these systems are currently not well described [16]. One recent study has found that GBS encodes as many as 21 TCRS [17]. Established GBS TCRS include DltR/S, which maintains constant levels of ᴅ-alanylation in GBS LTA [18]; RgfA/C, which represses the expression of C5a peptidase [19]; CovR/S global regulatory system, which controls the expression of multiple virulence factors [20]; LiaFSR, which regulates cell wall stress and pilus expression [21]; FspS/R, which regulates fructose-6-phosphate metabolism [17]; and CiaR/H, which promotes survival in the host intracellular environment [8].

We have demonstrated previously that the CiaR response regulator promoted GBS intracellular survival in phagocytic cells and brain endothelial cells [8]. Further, a GBS mutant deficient in *ciaR* exhibited increased susceptibility to killing by antimicrobial peptides, lysozyme, and reactive oxygen species [8]. GBS CiaR also contributed to overall virulence potential in a murine bacterial competition model of infection [8]. Thus, we hypothesize that CiaR regulation may impact GBS intracellular trafficking in BBB endothelium. Our previous studies compared the transcriptional profiles of WT GBS and the isogenic Δ*ciaR* mutant grown to log phase under identical conditions. Only one gene with a predicted function of purine and pyrimidine biosynthesis was upregulated more than twofold, while several genes were more dramatically down-regulated in the Δ*ciaR* mutant [8]. The most highly down-regulated gene, SAN_2180, encodes a conserved hypothetical protein, while the second most down-regulated gene, SAN_0039, belongs to the M23/M37 family of metallopeptidases, which catalyze the hydrolysis of nonterminal peptide linkages in oligopeptides or polypeptides [22]. Here we investigate the role of these CiaR regulated genes to GBS interaction with brain endothelial cells and to virulence potential.

## Materials and Methods

### Cell culture

The human brain microvascular endothelial cell line was kindly provided by Kwang Sik Kim (Johns Hopkins University) and cultured as previously described [23] in RPMI 1640 (VWR) containing 10% FBS, 10% Nuserum (BD Biosciences) and 1% nonessential amino acids (Life Technologies) at 37°C with 5% CO_2_.

### Bacterial strains and growth conditions

*Streptococcus agalactiae* (GBS) wild-type (WT) clinical isolate COH1 (serotype III) [24] and the isogenic Δ*ciaR* mutant described previously [8] were used for these studies. Isogenic mutants of COH1 in genes SAN_2180 and SAN_0039 were created by in-frame allelic replacement with a chloramphenicol resistance gene (*cat*) cassette using a previously described method [7]. Briefly, two flanking regions of target genes were amplified by PCR from COH1 genomic DNA, using 5'flank-F-2180: 5'- TAGCCATAACAGGAGATCCGACTA -3', 5'flank-F-0039: 5'- CCAACAGACTACTCAATCGCTTCAGC -3' and 5'flank-R-2180: 5'- TTTTATACCTCCCTTTCTCAA -3', 5'flank-R-0039: 5'- AGAATTAATATAATGAAGTGCTCAAACACTTG -3'; 3'flank-F-2180: 5'- TACTGATACAATACTAAGAA-3', 3'flank-F-0039: 5'-TCCAGTAAAGTGTGATATTATAGTCTC-3' and 3'flank-R-2180: 5'-TAGAGGAGGACACTGAATGACAAC -3', 3'flank-R-0039: 5'-CGTAGTCACAGGAACTGCTGG -3'. A *cat* cassette with complementary regions of target genes was amplified from as previously described [7] primers, Cat-F-2180: 5'-GAGAAAGGGAGGTATAAAAATGGAGAAAAAAATCACTGGATATACCACCGTTGA-3', Cat-F-0039: 5'-AGCACTTCATTATATTAATTCTATGGAGAAAAAAAT CACTGGATATACCACCGTTGA-3' and Cat-R-2180: 5'-TTCTTAGTATTGTATCAGTATTACGCCCCGCCCTGCCACTCATCGCAGTACTGTTGTA-3', Cat-R-0039: 5'-GTCCGAGACTATAATATCATTACGCCCCGCCCTGCCACTCAT CGCAGTACTGTTGTA-3'. The construct was then amplified with a pair of nested primers, Nest-F-XhoI-2180: 5'- CCGCTCGAGTCCCAGGAGCGACTAGTGTTTATG-3', Nest-F-XhoI-0039: 5'-CCGCTCGAGGATGATATTGAGACAGCTTG-3' and Nest-R-XbaI-2180: 5'- GCTCTAGAGGCTGGTATTGGGGACGGTATTTC-3', Nest-R-XbaI-0039: 5'- GCTCTAGACAGCGGCAACAGAAGCTGGT-3'; and then was cloned into the pHY304 vector. For complementation studies, full-length target genes were amplified using the following sets of primers, F-KpnI-2180: 5'- GGGGTACCGTATCGAATACTCACTT -3', R-SacI-2180: 5'- CGAGCTCCTCCATTATAGGAGGTT -3'; F-KpnI-0039: 5'- GGGGTACCTCATCAAGGTGAGTACTT -3' and R-SacI-0039: 5'- CGAGCTCATGAATCAATACCTCAAA -3' and cloned into pDCErm. Deletion mutant strains were transformed with the recombinant plasmids for generation of complemented strain. GBS strains were grown in Todd-Hewitt broth (THB, Difco) at 37°C and growth was evaluated by monitoring OD_600_. For antibiotic selection, 2 μg/ml chloramphenicol and 5 μg/ml erythromycin was incorporated in the growth medium when required. GFP-expressing GBS strains were created as previously described [9,12].

### Microscopy

Coverslips with GBS overnight culture were air-dried, heat fixed, and then subjected to a standard Gram stain protocol. Images were taken using a Zeiss upright microscope with an attached Axiocam Icc3 camera. For electron microscopy, 1mL (10^7^ CFU) of bacterial cells suspended in PBS was fixed in a cocktail of 2% gluteraldehyde and 1% osmium tetroxide in PBS for 10 minutes. The solution was then passed through a 0.4μm polycarbonate filter to collect bacterial cells and rinsed with 4mL of water. In order to dry the samples, the filters were taken through a series of increasing concentrations of ethanol (50, 75, 85, 95, 100%) before being placed in a Tousimis SAMDRI-790 critical point drying machine. The dried filters were mounted onto SEM sample stubs with a piece of double-sided carbon tape before applying a 6nm layer of platinum with a Quorom Q150ts high-resolution coater. Samples were viewed using an FEI FEG450 scanning electron microscope.

### *In vitro* infection assays

To determine the total number of cell surface-adherent or intracellular bacteria, hBMEC monolayers were grown to confluence in growth medium containing 10% FBS, 10% Nu-serum, and 1% non essential amino acids in 24-well tissue culture-treated plates. Bacteria were grown to mid-log phase and used to infect cell monolayers as described previously [23]. Briefly, hBMEC monolayers were incubated with GBS at 37°C with 5% CO_2_ for 30 min. To assess adherent bacteria, cells were washed five times with phosphate-buffered saline (PBS) to remove non-adherent bacteria, then trypsinized with 0.1 ml of trypsin-EDTA solution and lysed with addition of 0.4 ml of 0.025% Triton X-100 by vigorous pipetting. To assess intracellular bacteria GBS were incubated with hBMEC for 2h, cells were washed three times with PBS and 1 ml of media containing 100 μg/ml of gentamicin and 5 μg/ml of penicillin was added to each well to kill extracellular bacteria. Lysates were then serially diluted and plated on THB agar to enumerate bacterial colony-forming units (CFU). Bacterial adherence and invasion was calculated as (recovered CFU/original inoculum CFU)×100%. GBS intracellular survival experiments were performed as described above except that intracellular bacteria was enumerated at indicated time points.

### Immunofluorescence staining

GFP-expressing GBS strains were used to infect hBMEC monolayers. Following a 2h incubation and antibiotic treatment at indicated time points, hBMEC were fixed with 4% paraformaldehyde. Cells were then lysed with 0.1% Triton X-100, blocked with 10% FBS, and incubated overnight with antibodies (Cell Signaling Technology) to Rab5 (1:100), Rab7 (1:100), and LAMP1 (1:100). Cells were then washed and incubated with secondary antibodies (1:500) conjugated to Alexa-Fluor 594 (Life Technologies). Samples were visualized using a Zeiss Axiovert 200 inverted fluorescence microscope (Carl Zeiss). At least 100 cells per treatment were counted and all experiments were performed in triplicate.

### Lysosomal isolation

hBMEC were grown in 75 cm^2^ flasks at 37°C with 5% CO_2_ until confluence was achieved, and subsequently infected with WT and mutant strains of COH1 GBS at an MOI = 10 for 2 hours. After infection, cells were incubated with media containing penicillin (5μg/mL) and gentamycin (100μg/mL) for either 1 or 12 hours to eliminate extracellular bacteria. Cells were then washed with DPBS and subjected to lysosomal isolation using the Lysosomal Enrichment kit for Tissue and Cultured Cells according to manufacturer’s instructions (Thermo-Fisher). Briefly, ~200mg of cells were harvested with trypsin and centrifuged for 2 min at 850 × g. Lysosome enrichment reagent A containing a protease inhibitor cocktail (CalBioChem) was added to pelleted cells and subjected to a 2 min incubation on ice. After incubation, cells were then sonicated 15 times to lyse the cells and Lysosome enrichment reagent B containing a protease inhibitor cocktail was then added to the cells. Cells were then centrifuged for 10 min at 500 × g at 4°C. The supernatant was then collected and the final concentration was altered to 15% with OptiPrep Cell Separation Media. The samples were then loaded on discontinuous OptiPrep gradients varying from 30%, 27%, 23%, 20% to 17% in a 13.2 mL ultracentrifugation tube (Beckman-Coulter) and centrifuged in a SW 41 Ti rotor at 145,000 × g for 2 hours at 4°C. After centrifugation, the lysosomal fraction was isolated from the top gradient and washed using 2 volumes of DPBS in a microcentrifuge at 17,000 × g for 30 min at 4°C to remove OptiPrep media. Lysosomal pellets were then washed with Gradient Dilution Buffer at 17,000 × g for 30 at 4°C. Pellets were then re-suspended in 0.1% Triton X‒100 and plated on Todd Hewitt Agar to enumerate bacterial CFU. For lysotracker staining, pellets were re-suspended in PBS and stained with Lysotracker Red (Life Technologies) for 15 minutes and imaged using a Zeiss Axiovert 200 inverted fluorescence microscope (Carl Zeiss).

### *In vivo* competition assay

Animal experiments were approved by the Institutional Animal Care and Use Committee at San Diego State University (protocol APF 13-07-011D) and performed using accepted veterinary standards. Animals are housed 4 mice/cage per NIH standard space requirement in Micro-Isolater cages with contact bedding. The light cycle is 12/12 (light from 6am-6pm) and cages are changed 3 times/week. Mice are fed a standard rodent diet (Purina) using a free feed system with fresh food added weekly. During the experiment animals were monitored visually at least twice a day for signs of disease such as ruffled fur, lethargy or agitation and moribund appearance. Those animals showing the first signs of disease will be monitored a minimum of four times a day for worsening signs. Animal suffering from any of the symptoms: severe lethargy or agitation, moribund appearance, failure to right oneself after 5 seconds, may be defined as moribund and will be humanely sacrificed prior to the experimental endpoint. The method of euthanasia used was an overdose of CO_2_ followed cervical dislocation. In our studies no animals died prior to the experimental endpoint. 8-week-old male CD1 mice (Charles River Laboratories) were injected intravenously with 2×10^8^ bacteria at a 1:1 ratio of WT and either one of the isogenic mutant strains. After 72 hours, mice were euthanized and blood and brain were collected to enumerate bacterial CFU. PCR was performed to confirm the presence or absence of targeted genes on recovered CFU. Primers 2180_F: 5'-AGAGCACGTTATCCTTTCGCT-3' and 2180_R: 5'-TCCGCCAAAACGTGCAACAT-3'; and primers 0039_F: 5'- GAGCCAACTTTTCTTGGATGAC-3' and 0039_R: 5'- ACTAGATTGATTCTGTACAGGA-3' were used for screening. Experiments were repeated twice (5 mice/group).

### Statistical analysis

GraphPad Prism version 6.0 was used for statistical analyses and statistical significance was accepted at *p <* 0.05 *, *p<* 0.05; **, *p<* 0.005; ***, *p<* 0.0005; ****, *p<* 0.00005).

## Results

### Characterization of CiaR regulated genes

**Fig 1:**
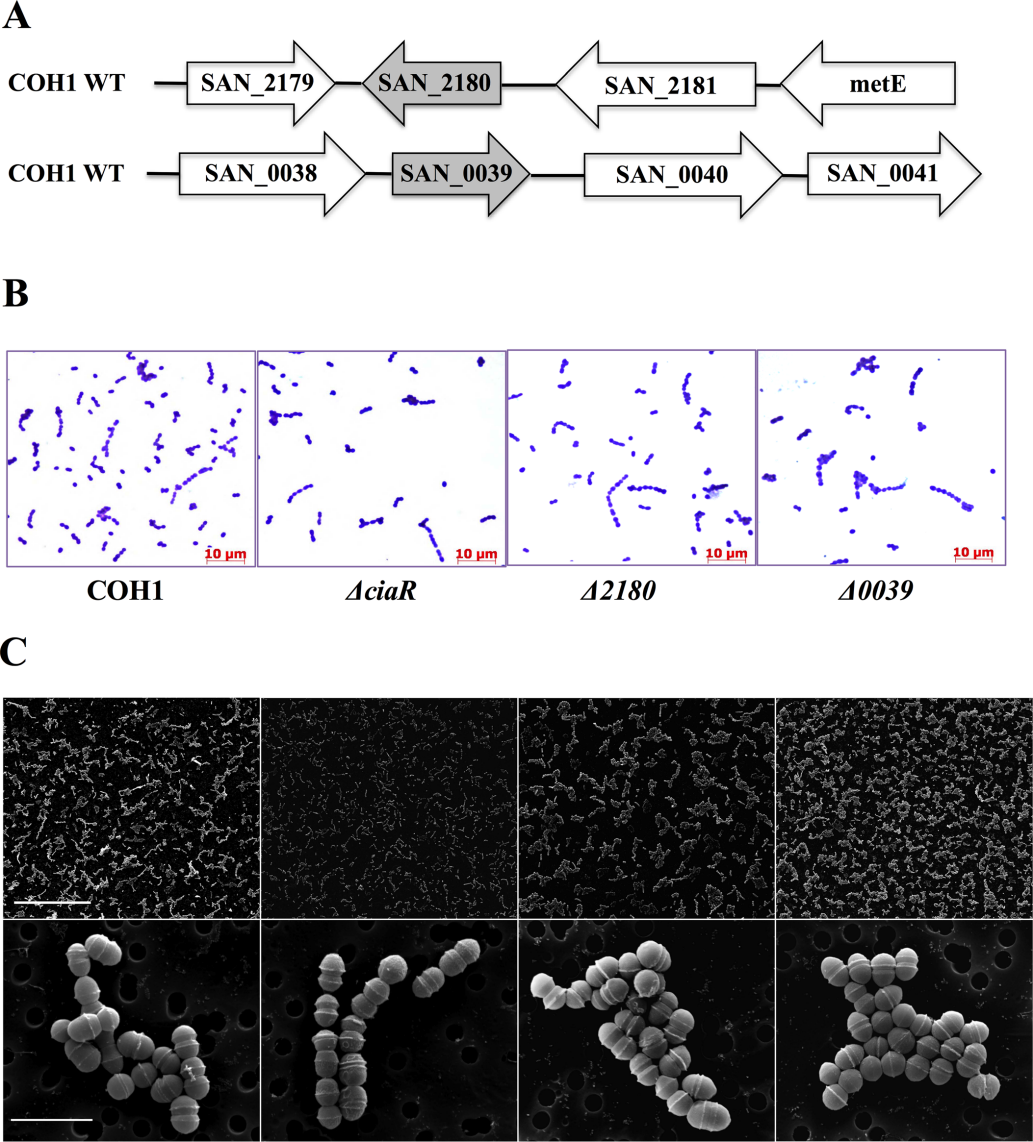
Morphology of GBS WT and mutant strains. A. Diagrammatic representation of the genetic locus surrounding SAN_2180 and SAN_0039 in the WT COH1 strain. Analysis of WT, Δ*ciaR*, Δ*2180* and Δ*0039* mutant strains by Gram staining (scale bar = 10 μm), B, and Scanning electron microscopy (top panel scale bar = 50 μm; bottom panel scale bar = 2μm), C.

Our data indicate that the response regulator CiaR may play a role in survival and trafficking within brain endothelial cells. Thus, we hypothesize that CiaR-regulated genes may impact GBS intracellular survival and the efficient trafficking of GBS through brain endothelium. Previous microarray analysis of the WT and Δ*ciaR* mutant identified the two most highly regulated genes, SAN_2180 and SAN_0039 [8] (Fig 1A). To characterize the impact of SAN_2180 and SAN_0039 on GBS interaction with human brain microvascular endothelial cells (hBMEC) and virulence, we generated isogenic knockout strains using in-frame allelic substitution of either gene with a chloramphenicol acetyltransferase (cat) resistance cassette using a method described previously [7] and as described in Materials and Methods. Both constructed mutants exhibited similar growth rates in THB compared to the WT parental strain (data not shown) and similar morphology as observed by Gram stain and electron microscopy (Fig 1B and 1C), although the Δ*ciaR* mutant appeared to grow in shorter chains.

**Fig 2:**
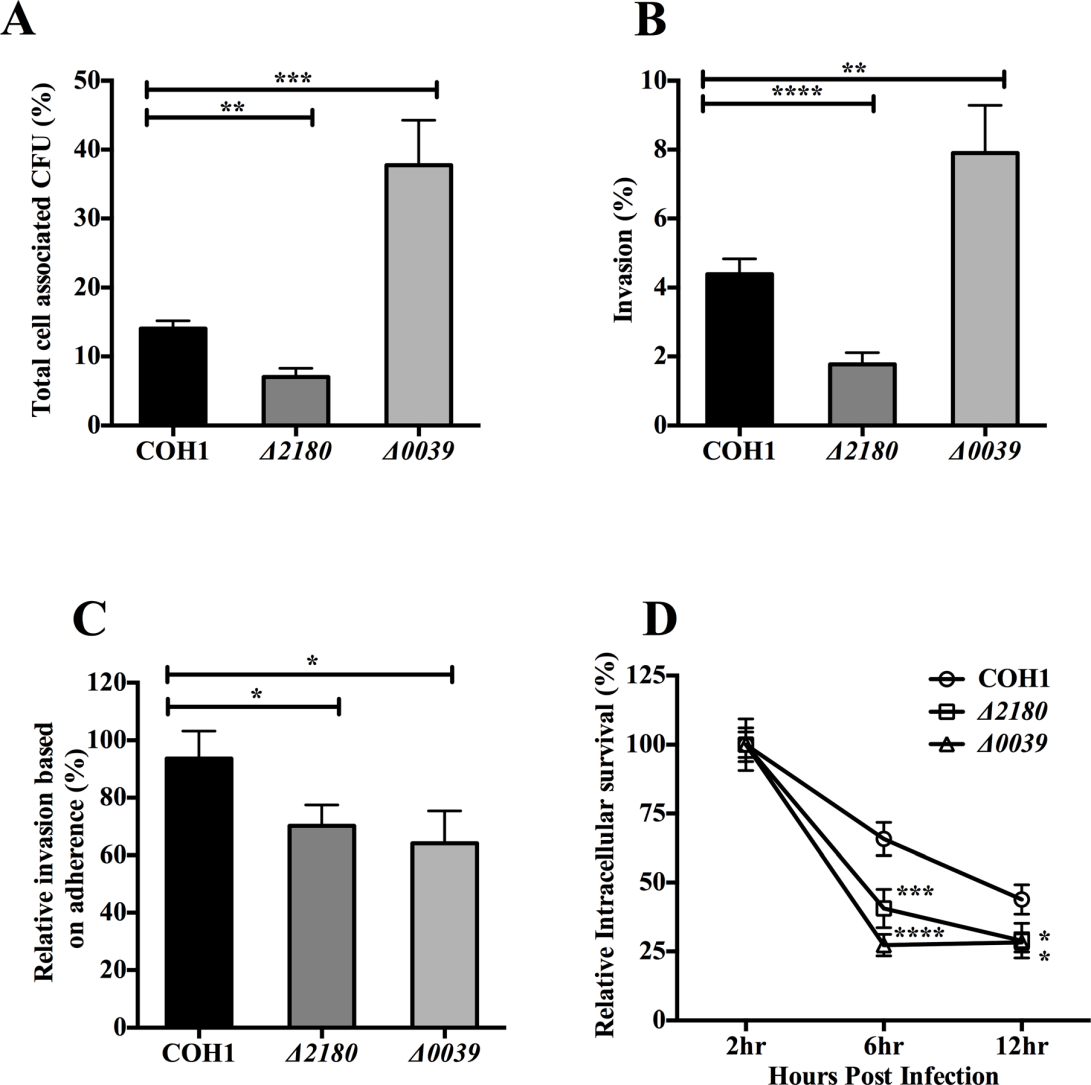
Adherence, invasion, and intracellular survival of GBS in brain endothelial cells are influenced by SAN_2180 and SAN_0039. A. Adherence to hBMEC by WT and mutant strains (MOI = 1). B. Invasion of hBMEC by WT and mutant strains (MOI = 1). C. Relative percentage of the hBMEC-associated GBS, WT and mutant strains, that had invaded the intracellular compartment (MOI = 1). D. Intracellular survival of WT and mutant GBS strains in hBMEC over time. Statistical analysis performed was a Two-way ANOVA with a Bonferroni’s multiple comparisons test and the data represents mean ± S.D. *p <* 0.05 *, *p <* 0.0005 ***, *p <* 0.00005 ****.

We have previously shown that the Δ*ciaR* mutant exhibited decreased survival in hBMEC [8], thus we characterized the interactions of the Δ*2180* and Δ*0039* mutants with hBMEC, specifically the adherent and invasive capabilities as well as the ability to survive and persist intracellularly. The Δ*2180* mutant exhibited a ~2-fold and significant decrease in hBMEC adherence and invasion compared with the WT parent strain (P < 0.005 and P < 0.00005, respectively) (Fig 2A and 2B). Interestingly, the Δ*0039* mutant displayed increased adherence and invasion into hBMEC (Fig 2A and 2B), however, when calculating the percentage of the hBMEC-associated GBS that had invaded the intracellular compartment both mutants exhibited decreased invasive capability compared to the WT strain (Fig 2C). These data indicate that both SAN_2180 and SAN_0039 contribute to GBS uptake into hBMEC. To examine whether these genes impact intracellular survival, we infected hBMEC with WT and mutant strains for 2 hours, incubated with extracellular antibiotics and at 2, 6, and 12 hours post antibiotic treatment, the intracellular pool was quantified as described in Materials and Methods. The percent of invasive bacteria recovered over time is shown relative to the first time point for each strain (Fig 2D). We observed a gradual decrease in intracellular WT bacteria over time as we have demonstrated for GBS in hBMEC previously [7]. However, the level of intracellular organisms over time for each of the mutant strains was significantly less compared to the WT strain (Fig 2D).

### Characterization of GBS intracellular trafficking in brain endothelial cells

**Fig 3:**
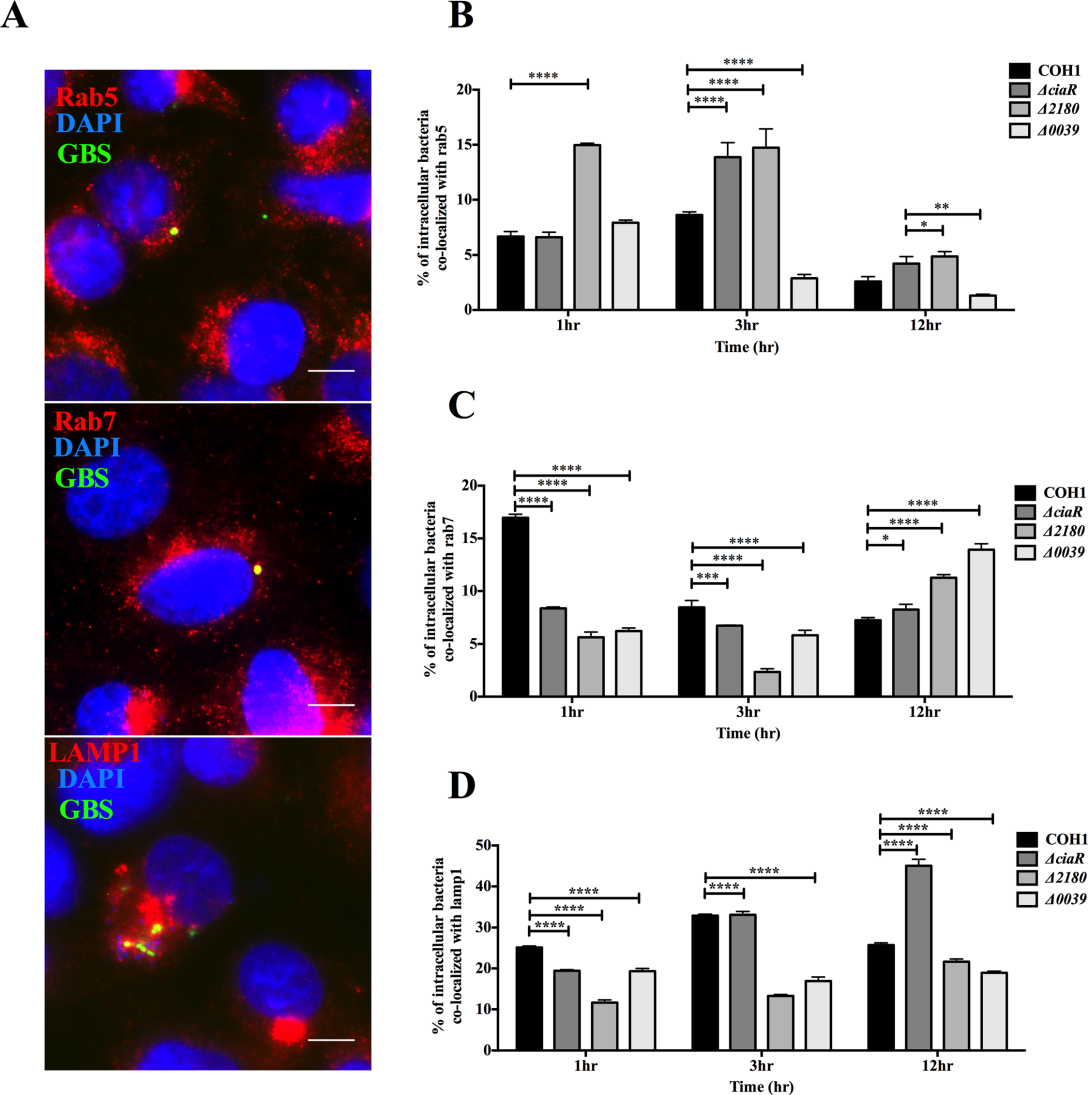
CiaR regulation influences GBS intracellular trafficking in brain endothelial cells. A. hBMEC monolayers were infected with WT COH1 GFP expressing GBS for 2 hours (MOI = 10) and then stained with antibodies to Rab5, Rab7, and LAMP1 following the indicated time points as described in Materials and Methods. Representative images of triplicate experiments demonstrate co-localization (yellow) of GBS (green) with each marker (red), Scale Bar, 5μm. B, C, D. Percentages of co-localization of Rab5, Rab7, and LAMP1 with GFP expressing GBS WT and mutant strains after various time points post infection during antibiotic treatment. At least 100 cells containing intracellular GBS were counted for each time point in triplicate. Statistical analysis performed was a Two-way ANOVA with a Bonferroni’s multiple comparisons test and the data represents mean ± S.D. *p <* 0.05 *, *p <* 0.0005 ***, *p <* 0.00005 ****.

Efficient trafficking of bacteria through endothelial barriers is a hallmark of the development of a multitude of vascular diseases. Endocytic trafficking consists of labeling by Rab GTPases, small guanine nucleotide binding proteins responsible for vesicular trafficking of cargo, and selectively transporting intracellular pathogens to the lysosome [13,25]. Rab5 is a monomeric GTPase known to be involved in early endocytic trafficking, while Rab7 acts later in the endocytic pathway to regulate lysosomal fusion [26]. Rab5 and Rab7 specifically label early and late endosomes, respectively, and subsequently traffic cargo or pathogens to the lysosome, which is labeled by lysosomal associated membrane protein 1 (LAMP1). We investigated the association of WT GBS and the Δ*ciaR*, Δ*2180* and Δ*0039* mutants with Rab5, Rab7, and LAMP1 labeled compartments. Infection of hBMEC with GFP-GBS strains was carried out for 2h as described in Materials and Methods, then at various time points post antibiotic treatment, cells were processed and stained for endosomal and lysosomal markers. Representative images following WT GBS infection 1h post antibiotic treatment show GBS localizing with each marker during the infection time (Fig 3A). To quantify co-localization over time, for each time point, we counted triplicate biological samples, at least 100 cells with intracellular bacteria and evaluated Rab5, Rab7, and LAMP1 localization with GBS WT and mutant strains (Fig 3B–3D). At early time points there were either similar amounts of WT and Δ*ciaR* mutant GBS that localized with endosomal and lysosomal-labeled vesicles or in some cases there were higher amounts of WT GBS. However at later time points, significantly more of the Δ*ciaR* mutant was associated with both Rab and LAMP1 markers. These findings demonstrate that GBS associates with vesicles involved in the endocytic pathway, with 25–30% of intracellular bacteria localizing with the lysosome.

Generally the *Δ2180* mutant strain exhibited similar localization with Rab5 and Rab7 positive vesicles as the *ΔciaR* mutant, except at the early time point where we observed that the *Δ2180* mutant co-localized more with rab5 (Fig 3B and 3C). It is also notable that the WT strain co-localized more than other strains with rab7 and LAMP1 positive vesicles at the early time point. However, both *Δ2180* and *Δ0039* mutants co-localized less with LAMP1 positive cells when compared to WT and the *ΔciaR* mutant strain, particularly at later time points (Fig 3D). Thus, overall CiaR regulation and specific regulated genes may influence GBS trafficking through endocytic compartments in different ways.

### Recovery of GBS from lysosomes isolated from brain endothelial cells

**Fig 4:**
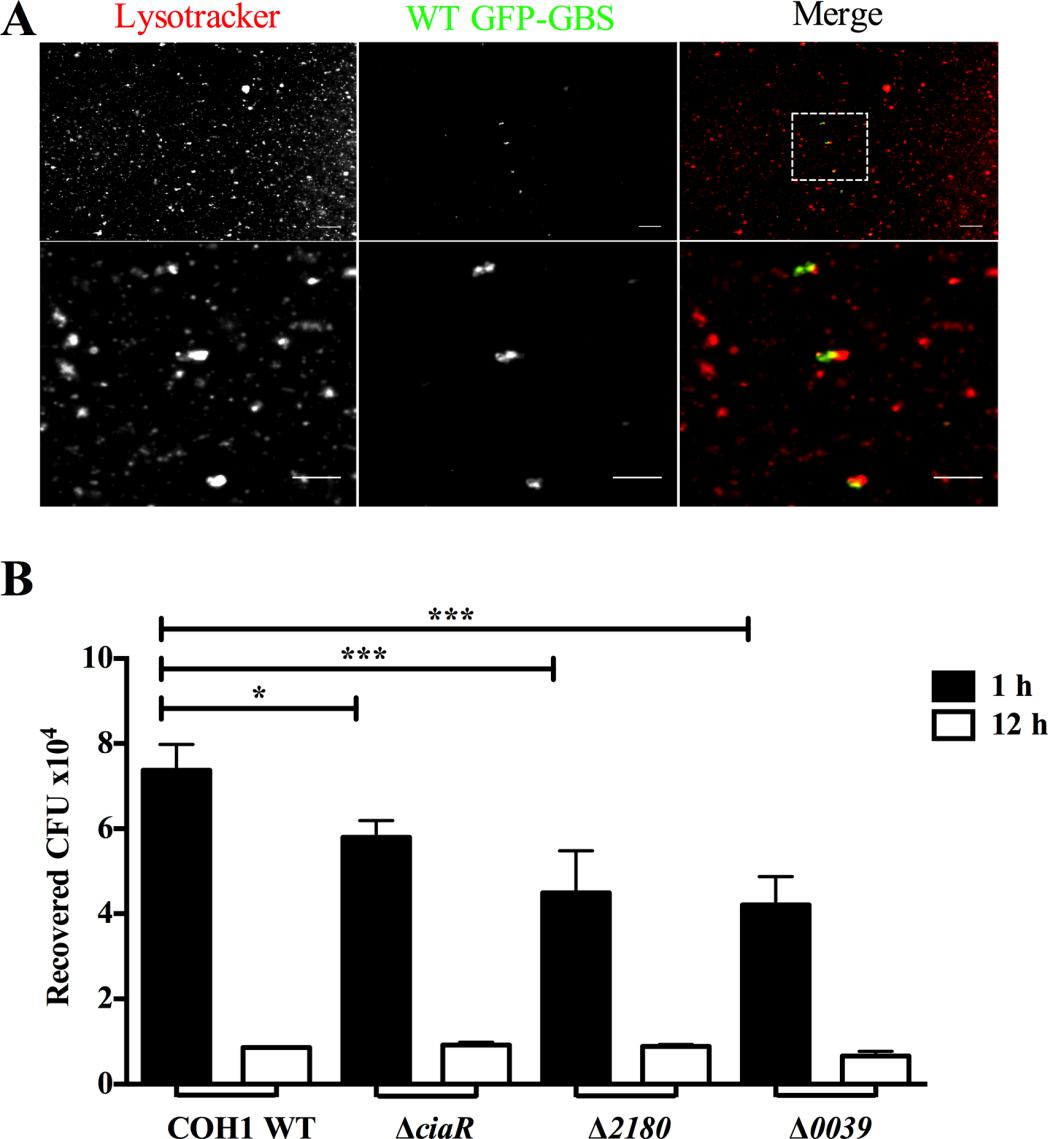
GBS recovery from Lysosomes. A. Isolated lysosomes infected with WT COH1 GFP expressing GBS were subjected to staining with Lysotracker Red (0.5μM) and visualized using fluorescence microscopy. Scale Bar, 5 μm B. hBMEC were infected with WT or mutant GBS strains for 2 hours (MOI = 10) and subjected to lysosomal isolation by differential centrifugation after 1 or 12 hours post antibiotic treatment. Lysosomal pellets were plated to enumerate the amount of recovered viable CFU. Statistical analysis performed was a One-way ANOVA with a Tukey’s multiple comparisons test and the data represents mean ± S.D. *p <* 0.05 *, *p <* 0.005 **.

Our results suggest that GBS may regulate genes to prevent or limit endocytic trafficking to the lysosome. Although we observed that there was actually less co-localization of both Δ*2180* and Δ*0039* mutants with lysosomal markers at later time points, it is possible that these mutants are still readily trafficked to acidic compartments, but exhibit increased sensitivity to lysosomal killing. Thus, we investigated whether we could recover viable intracellular GBS from lysosomes isolated from brain endothelial cells. We used a lysosomal enrichment protocol that employs differential centrifugation to enrich for lysosomes based on size and density. Following hBMEC infection with WT and mutant GBS strains for 2 hours, cells were treated with antibiotics, and lysosomes were subsequently isolated at early (1 hour) and late (12 hours) time points. Western blot analysis was performed to confirm that recovered lysosomes were positive for LAMP1 (data not shown). We first used Lysotracker, which stains acidic vesicles, and found that WT GBS could be visualized within acidified lysosomes (Fig 4A). Lysosomes were also lysed in 0.1% Triton X-100 and lysates plated on THB agar to enumerate viable bacteria. Markedly more WT GBS was recovered from the lysosome fraction than any of the mutant strains at the early time point post infection (Fig 4B). Furthermore, fewer viable GBS was recovered from lysosomes at the later time point. These data further indicate that GBS does indeed traffic to the lysosome and that CiaR, SAN_2180, and SAN_0039 contribute to initial GBS trafficking and survival.

### CiaR regulated genes contribute to GBS virulence

**Fig 5:**
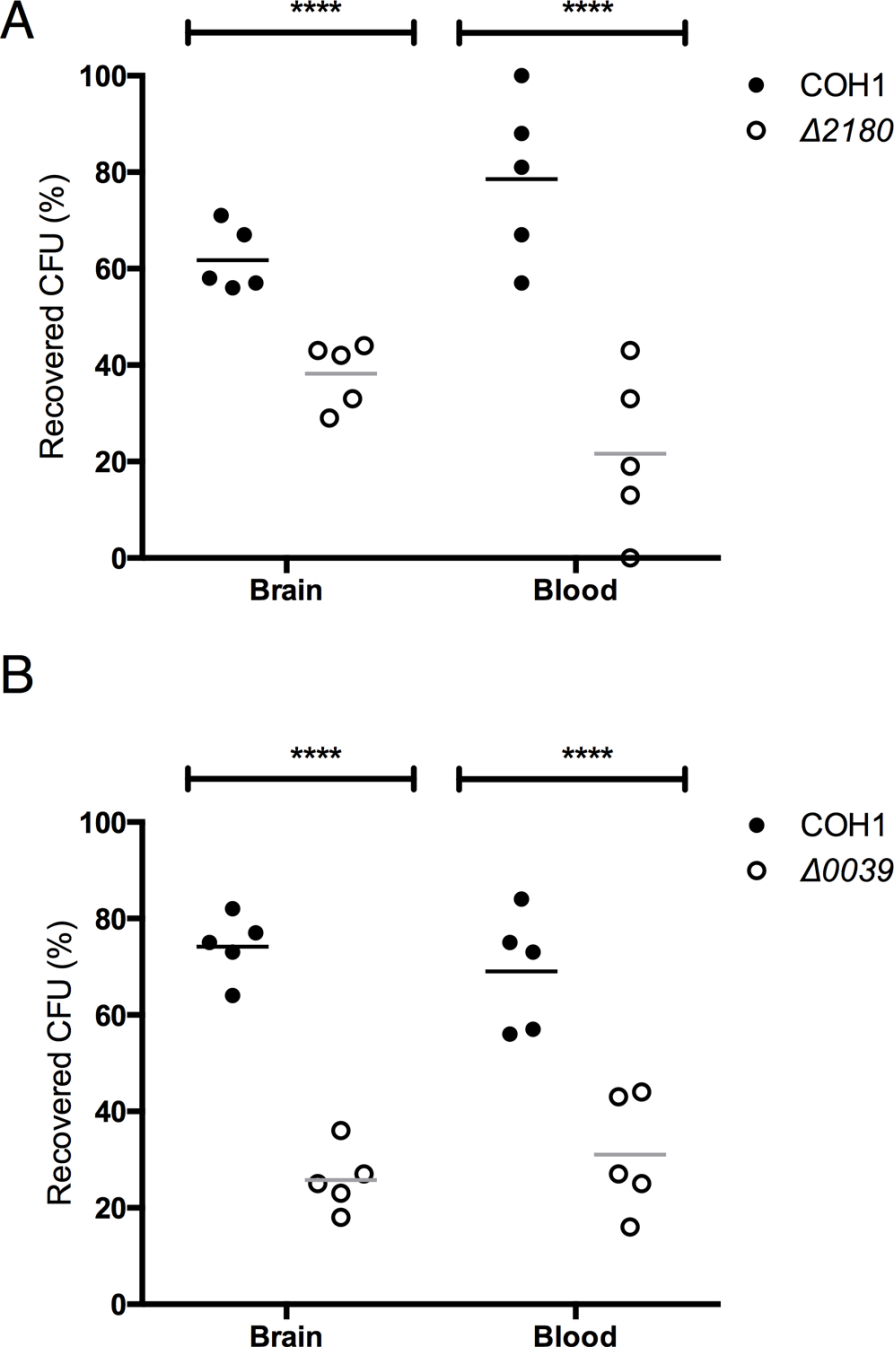
GBS SAN_2180 and SAN_0039 contribute to overall bacterial virulence. The recovered bacterial CFU in blood and brains were analyzed 72 h after intravenous injection with equal amounts of WT and mutant GBS strains into CD1 mice. Bacteria were enumerated on THA plates with serial dilutions and the bacterial colonies from the same dilution were distinguished by colonies PCR between WT and mutant strains. A. The percentage of recovered CFU in blood and brains from WT and the Δ2180 mutant strain. B. The percentage of recovered CFU in blood and brains from WT and the Δ0039 mutant strain. Statistical analysis performed was an unpaired t-test and the data represents mean ± S.D. *p <* 0.00005 ****.

We have demonstrated previously that CiaR promotes bacterial fitness and overall virulence in a mouse model of GBS infection [8]. To similarly examine whether the CiaR regulated genes contribute to virulence *in vivo*, we employed the same bacterial competition model as described previously [8,27]. Mice were challenged intravenously with equal amounts (2 × 10^8^ CFU) of WT COH1 and either Δ*2180* or Δ*0039* isogenic mutants. At the experimental end point (72h), mice were euthanized and blood and brains were collected for the enumeration of surviving bacteria. PCR-based screening was used to distinguish between the WT and mutant strains. Data is expressed as the percentage of WT or mutant GBS recovered compared to total recovered CFU. Consistently more WT GBS than the Δ*2180* or Δ*0039* mutant strains were recovered from the blood and brain (Fig 5A and 5B). This is consistent with previous results with the Δ*ciaR* mutant [8], and suggests that both CiaR regulated genes contribute to bacterial fitness and virulence.

## Discussion

Penetration of the BBB likely requires GBS to invade microvascular endothelial cells and transcytose through cells, exiting basolaterally to breach the CNS. It is known that GBS can persist within brain endothelial cells for up to 24 hours post infection, however, there is no net increase in bacterial replication, and the intracellular pool actually decreases over time [7,11]. This is likely due to the ability of the host cell to limit GBS intracellular growth by using various forms of antibacterial defense including transit to the lysosomal compartment for subsequent degradation. We observed that intracellular WT GBS readily acquired markers of endosomal maturation, as GBS associated with early and late endosomes, and approximately 25–30% of intracellular organisms localized with acidified lysosomal vesicles (Fig 3). Our results presented here suggest that GBS may use the response regulator, CiaR, to prevent endocytic trafficking to the lysosome. A GBS mutant deficient in CiaR exhibited increased localization with vesicles in the endocytic pathway including Rab5, Rab7 and LAMP1 positive cells. Rab GTPase modifications have been identified during bacterial infection and Rab5 modulation by numerous pathogens has proven to be an efficient strategy to promote intracellular replication or persistence [28]. Additionally, bacteria such as *Helicobacter pylori* and *Mycobacterium bovis* may modulate Rab7 endosomal maturation in order to establish a protective intracellular niche during infection [29,30]. Interestingly, following infection with the Δ*ciaR* mutant less viable bacteria was recovered from lysosomes isolated from hBMEC. This is likely due to the increased sensitivity of the Δ*ciaR* mutant to the hostile environment of the phagolysosome, namely antimicrobial peptides and reactive oxygen species, which we have demonstrated previously [8]. The reduced recovery of the Δ*2180* and Δ*0039* mutant strains from lysosomes likely also reflects an increased sensitivity to lysosomal killing as we observed these mutants, like Δ*ciaR*, were more sensitive to antimicrobial peptides, lysozyme, and H_2_O_2_ (data not shown).

While GBS is not thought of as a classic intracellular pathogen, GBS survival in phagocytic cells has been reported [31,32]. Most research has focused on understanding the virulence factors responsible for GBS persistence in human macrophages and neutrophils. The pore forming β-hemolysin/cytolysin (β-h/c) encoded by *cylE* is a major virulence factor contributing to GBS disease progression [3]. Interestingly, *cylE* deletion results in the loss of β-h/c activity and the carotenoid pigment. It has been shown that *cylE* contributed to enhanced GBS survival within phagocytes that was attributed to the ability of carotenoid to shield GBS from oxidative damage [33]. However, other reports suggest that the ß-h/c did not impact intracellular survival in macrophages (Cumley *et al*., 2012) or even that the absence of the β-h/c enabled increased GBS survival in professional phagocytes [34]. It is possible that these results reflect differences in GBS strains, β-h/c production, and/or host cells and cell lines, and requires further investigation. Other GBS cell associated factors reported to impact survival in phagocytic cells include pili [27] and the capsule polysaccharide [35,36]. An additional TCRS, the CovR/S global regulator that regulates many genes including *cylE*, has also been shown to be required for intracellular survival in macrophages [37]. That study suggests that CovR/S mediates a transcriptional response stimulated by the acidic environment in the phagolysosome that mediates survival [37]. Less is known about GBS survival in epithelial or endothelial cells. Interestingly, we have previously infected hBMEC with a GBS Δ*covR* mutant to assess bacterial uptake and survival, and were not able to recover viable Δ*covR* bacteria from the intracellular compartment [20]. However, at this point we cannot conclude whether CovR regulation is required for GBS invasion into brain endothelial cells, or if it regulates intracellular survival.

Two-component regulatory systems allow bacteria to adapt to changing environmental conditions. CiaR/H is not fully characterized in GBS, but it has been linked to stress tolerance and host defense resistance similar to the role of CiaR/H in *Streptococcus mutans* [38] and *Streptococcus pnuemoniae* [39]. Interestingly, the *S*. *pneumoniae* CiaR homologue has also been described to be involved in β-lactam resistance and lytic capabilities [40,41]. CiaR-deficient GBS displayed decreased intracellular survival in neutrophils, macrophages, and brain microvascular endothelial cells and was more susceptible to killing by antimicrobial peptides and reactive oxygen species, suggesting CiaR/H as a vital element for environmental stress tolerance [8]. Previously, our group identified a subset of genes that are down-regulated in a CiaR- deficient mutant. One gene, SAN_0039, encodes a putative metallopeptidase exhibiting a high degree of homology (70% similarity, 56% identity for 91%protein coverage) to a protein called Zoocin A (*zooA*) [8]. Zoocin A is produced by *S*. *zooepidemicus* (Group C *Streptococcus*) which has a bacteriolytic effect on several other Streptococcal species [42]. Zoocin A has two functional domains, an N-terminal catalytic domain and a C-terminal substrate-binding or target recognition domain [43,44]. Zoocin A has been determined to act as a ᴅ-alanyl-l-alanine endopeptidase which hydrolyses the cross bridge of peptidoglycan of certain *Streptococcus* species [45]. Utilization of peptidoglycan hydrolases for both peptidoglycan rearrangement and pathogenicity in host cells has been described in several bacteria. *S*. *pneumoniae*, *Listeria monocytogenes*, and *Staphylococcus aureus* employ differential acetylation strategies to obtain resistance to lysozyme [46–48]. Another peptidoglycan hydrolase, known as IspC, has been identified in *L*. *monocytogenes* as being essential for virulence *in vivo*, and crossing the blood-cerebrospinal fluid barrier [49]. The attenuated virulence of an IspC deficient mutant may be partly due to the reduced surface expression or display of other known or putative virulence factors [49]. We observed that while the GBS Δ*0039* mutant exhibited increased adherence to hBMEC, invasion into cells was reduced, suggesting a defect in a surface factor(s) that modulates bacterial uptake. Future studies using proteomic analysis of GBS WT and the *Δ0039* mutant strain will be of interest to determine if the GBS peptidoglycan hydrolase contributes indirectly to host cell interaction and virulence by modulating surface targeting mechanisms that affect other GBS factors. We should note that overexpression of the 0039 gene was toxic to GBS making complementation experiments impossible. This is consistent with what has been observed for Zoocin A (R. Simmonds, personal communication).

Another GBS gene, SAN_2180, was the most highly down-regulated gene in the Δ*ciaR* mutant [8]. Characterization of a Δ*2180* mutant demonstrated that this gene contributes to bacterial uptake, a phenotype that was complemented by reintroducing the WT gene back into the Δ*2180* mutant (data not shown). The Δ*2180* mutant also exhibited decreased survival within brain endothelial cells as well as decreased virulence potential *in vivo*. Like the Δ*ciaR* mutant, the Δ*2180* mutant more readily localized with endosomal and lysosomal marked cells, but was not readily isolated from lysosomes even at early times points likely due to an increased sensitivity to antimicrobial factors. Thus this factor may be the primary CiaR regulated gene responsible for the observed phenotype of the Δ*ciaR* mutant, although future studies with a double mutant strain would help clarify this. Protein sequence analysis using BLAST predicted that the SAN_2180 protein belongs to the proteins of unknown function family, DUF1003, but shares homology (60% similarity, 42% identity for 93% protein coverage) to a protein in *Lactococcus lactis* involved in acid tolerance and multistress tolerance [8,50]. Additionally, the SAN_2180 protein sequence is homologous to cyclic nucleotide-binding proteins present in other *Streptococcus* species such as *Streptococcus urinalis* (84% identity for 100% protein coverage), *Streptococcus parasanguinis* (69% identity for 98% protein coverage) and *Streptococcus gallolyticus* (60% identity for 98% protein coverage). Cyclic nucleotide-binding proteins are important for binding intracellular messengers such as cyclic AMP [51]. Modulation of host cAMP levels has been proven to be a novel bacterial mechanism to engage inflammatory responses and disease progression [52]. However, further characterization of the SAN_2180 encoded protein is needed to elucidate specific mechanisms responsible for its role in GBS invasion and intracellular survival.

In summary our data suggest that GBS may modulate gene expression through the TCRS CiaR/H to promote intracellular survival. This may impact trafficking to the lysosome as at later time points we observed only 25% of intracellular WT bacteria localizing with LAMP1 positive vesicles compared to 45% in the absence of CiaR regulation. Interestingly, we have not observed GBS free in the cytoplasm of hBMEC, even at later time points [12], suggesting that surviving GBS likely traffics through endosomes in brain endothelial cells to promote transcytosis across the BBB. However further experimentation is required to fully characterize the fate of all GBS-containing endosomes and how a percentage of GBS bacteria may avoid the lysosomal compartment and transit through the brain endothelium in order to breech the CNS. Our data suggest that the specific CiaR regulated genes, SAN_2180 and SAN_0039, may not independently explain the phenotype of the CiaR deficient mutant, but still provide interesting new bacterial targets that may further inform the mechanisms of BBB penetration as well as the development of preventative therapies.
